# How path dependency manifests in flood risk management: observations from four decades in the Ennstal and Aist catchments in Austria

**DOI:** 10.1007/s10113-023-02029-y

**Published:** 2023-01-30

**Authors:** Sebastian Seebauer, Thomas Thaler, Susanne Hanger-Kopp, Thomas Schinko

**Affiliations:** 1 LIFE Institute for Climate, Energy Systems and Society, JOANNEUM RESEARCH Forschungsgesellschaft Mbh, Graz, Austria; 2 Institute of Mountain Risk Engineering, University of Natural Resources and Life Sciences, Vienna, Austria; 3 Population and Just Societies Program, International Institute for Applied Systems Analysis, Laxenburg, Austria; 4 Department for Environmental Systems Science, ETH Zürich, Zurich, Switzerland

**Keywords:** Spatial planning, Risk management, Water management, Natural hazards, Flood protection, Transformation

## Abstract

Path dependency occurs when a contingent event predetermines what further steps can be taken and self-reinforcing mechanisms lock-in any further development on a sub-optimal trajectory. Path dependency is a prominent concept in the adaptation pathways literature, but insufficiently defined and operationalised. The present paper empirically tracks all constitutive elements of path dependency for four decades of flood risk management (FRM) in two alpine mountain regions in Austria, the Ennstal and Aist river catchments, using a mixed-methods approach. FRM governance has a critical role whether decisions lead to path dependency. Lock-in manifests not just in technical structures, but also in inertia of incumbent actor coalitions and management paradigms. Sub-optimality is hard to assess for lack of clearly defined protection targets; however, it appears in the ways that structural measures are implemented—too little, too late or with negative impacts on nature conservation. Past floods do not qualify as contingent events, as they have not fundamentally changed FRM practice. By contrast, technological and institutional shifts over longer periods, such as digital hazard maps and EU directives, have gradually reoriented FRM strategies. Institution-based self-reinforcing mechanisms are more prevalent than technology-based self-reinforcing mechanisms. Established actor coalitions combined with institutional density illustrate how those in charge uphold a path to defend their position, power and resources. Our recommendations for how to overcome path dependency in FRM governance are: encourage niche experiments, link FRM more closely with climate change adaptation, revise the national policy framework towards polycentric governance approaches and improve professional training.

## Introduction

The accumulation of extreme flood events in recent years, such as the Kenya and Uganda floods in 2020 or Ahrweiler in Germany and Henan Flood in China in 2021, and projections of further increase in frequency and magnitude of flood events due to climate change call for long-term, strategic planning in flood risk management (IPCC 2021). Impacts of a warmer climate heavily affect exposed buildings in floodplains worldwide (Tedesco et al. 2020). Climate impacts are exacerbated by current land uses and land cover (IPCC 2018) as well as by policy decisions and socio-demographic changes (Clar et al. 2021a). Conversely, lack of designated areas and different socio-cultural interests still increase the pressure to construct more buildings in the floodplain (Kundzewicz et al. 2014; Rajib et al. 2021). To respond adequately to these current and future challenges, policymakers need to find innovative and transformative solutions to manage increasing flood risk (Thaler et al. 2019).

Nonetheless, decisions in flood risk management (FRM) often fall back on a narrow set of established problem-solving strategies, such as technical risk mitigation measures, emergency response or damage compensation. Decisions are often taken in a quick and ad hoc manner in the aftermath of flood events within the current FRM paradigm and focus on bounce-back to restore the pre-disaster situation instead of bounce-forward towards a climate-resilient society, bridging the still disconnected policy domains of disaster risk reduction, climate change adaptation and sustainable development (Leitner et al. 2020; Slavikova et al. 2021). The result often does not alleviate the vulnerability of the affected community (Wisner et al. 2004; Mika and Kelman 2020). Current decision processes tend to replicate past strategies and neglect alternative options, resulting in what is often called path dependency.

Path dependency is most frequently applied with regard to climate change adaptation in general (e.g. Thomsen et al. 2012; Pauw and Pegels 2013; Wise et al. 2014; Barnett et al. 2015; Hogarth and Wójcik 2016; Nair and Howlett 2016; Sheller and León 2016; Hölscher et al. 2019; Lassa 2019; Mummery and Mummery 2019). Path dependency is also applied to more specific policy areas such as infrastructure management (e.g. Ulibarri and Scott 2019; Matthews et al. 2015; Chester and Allenby 2019), energy efficiency measures (e.g. Smith and Brown 2014) and water management (e.g. Burnham et al. 2016). The adaptation pathways literature inherently addresses path dependency, as per definition they compare and sequence adaptation measures, and often considers multiple stakeholder perspectives (Werners et al. 2021; Hanger-Kopp et al. 2022).

However, while path dependency is prominent in academic and political debate, the pertinent literature defines the concepts underlying path dependency ambiguously, applies these only selectively to practical cases in FRM and tends to favour a technology-based over an institution-based perspective (Hanger-Kopp et al. 2022). FRM governance plays a central role in entering, remaining on or leaving paths (e.g. Gralepois et al. 2016; Wiering et al. 2017; Liefferink et al. 2018). For instance, in Austria, the focus of the present paper, a multi-level network of public institutions with dedicated missions and budgets, promotes technical flood mitigation measures, thereby precluding alternative solutions (Mochizuki et al. 2018).

The aim of the paper is thus twofold: first, we show empirically how theoretical concepts of path dependency manifest in the real world, comprehensively tracking all elements of path dependency at the local level where the effects of FRM practice are most tangible. To this end, we reconstruct four decades of FRM in two alpine mountain regions in Austria, the Ennstal and Aist river catchments. Thereby, we operationalise our previous conceptual work on adaptation pathways approaches (Hanger-Kopp et al. 2022). Second, we discuss what lessons can be learned and suggest recommendations for how to overcome path dependency in FRM governance. We cannot empirically establish prototypical path dependency in our study regions; rather, elements of path dependency appear more implicit and less clear-cut than theory might suggest. We demonstrate that FRM governance plays a critical role in all elements of path dependency, primarily as institution-based self-reinforcing mechanisms. Technological and institutional shifts, as well as niche experiments, may support a gradual reorientation of prevalent paths.

## Path dependency and flood risk management

There is limited academic literature exploring path dependency in an FRM context. Some studies may lead with path dependency, but rather explore change and thus do not provide any specific definition of the concept or its elements (e.g. Garrelts and Lange 2011); other literature focuses on a subset of elements (e.g. Gralepois et al. 2016, Wiering et al. 2017, Liefferink et al. 2018, Tellman et al. 2018, Parsons et al. 2019). Next, we discuss the constitutive elements of path dependency, complementing the definitions from the original literature from technology and policy studies with empirical findings from FRM research.

Path dependency goes well beyond a “history matters” approach. Indeed, it is seen “as a process that has the property of staying on a particular [trajectory], so that past decisions and contingent events pre-determine what further steps may be taken. [Under such circumstances] technologies, policies, or governance modes are locked-in [and] self-reinforcing mechanisms contribute to their reproduction and diminish the range of likely alternatives.” (Hanger-Kopp et al. 2022, p. 2).

From the multi-disciplinary literature on path dependency, several unifying characteristics and conditions of path dependency stand out: *Lock-in*, from a technical point of view, refers to a state where endogenous change is impossible, whereas, from an institutional point of view, it rather refers to a phase of minor or incremental change, as an absolute lock-in is unlikely (North 1990). In FRM, Parsons et al. (2019) find lock-in as a result from ingrained institutional arrangements wherein powerful actor coalitions preserve their interests, as well as a dominant discourse of human and technological supremacy in controlling the environment. Gralepois et al. (2016), Wiering et al. (2017) and Liefferink et al. (2018) use the term stability rather than lock-in when exploring flood risk governance in Belgium, France, the Netherlands and Poland over three decades.

*Sub-optimal outcomes*, that is, falling short of stated targets are another characteristic of path dependency. Sub-optimality is difficult to determine, particularly in an institutional context where different perspectives on optimality can be valid. Moreover, sub-optimality gives path dependency a negative connotation that is not necessarily justified, as path dependency can be productive if it leads to desired goals, and targets may change over time. We have not found FRM studies clearly addressing this element of path dependency, although Wiering et al. (2017) highlight new ideas and awareness of the sub-optimality of existing paths as important drivers of change. In FRM, sub-optimality is traditionally referred to as a negative ratio in cost–benefit considerations; these considerations should however also take intangible, social and non-monetary aspects into account (Penning-Rowsell et al. 2013; Thaler and Priest 2014; Babcicky et al. 2021).

*Contingent events* initiate a path-dependent trajectory, overriding the initial situation. Strictly speaking, contingent events are random and without antecedent causes, which makes them almost impossible to verify or falsify. A reasonable and practical operationalisation for contingent events could be a result “of circumstances that are unusual, surprising for the planning process and often not anticipated in a particular organizational, governance, and institutional setting” (Hanger-Kopp et al. 2022, p. 3). This creates some questions for practical applications such as in the present study. For example, can we consider flood events in general as contingent events? Ample evidence shows that disaster events provide windows of opportunity for large-scale change (Christoplos 2006; Birkmann et al. 2010; Kates et al. 2012; Sword-Daniels et al. 2015; Clar and Steurer 2019). Floods can actually be anticipated in risk modelling; yet, by definition, contingent events should be unpredictable and only marginally related to the historical development of the risk. Contingent events need not be limited to disaster events but may also include institutional rearrangements or technological and social innovations (Hanger-Kopp et al. 2022). Parsons et al. (2019) analyse path dependency in FRM in the Rangitāiki Plains of Aotearoa, New Zealand, over more than a century. This path started with the contingent events of indigenous dispossession and the marginalisation of Māori values in environmental governance and policy.

*Self-reinforcing mechanisms*—the most prominent condition of path dependency in the literature—are easier to identify also in the case of FRM. They refer to positive feedback or increasing returns that ensure the continuity of a path. Self-reinforcing mechanisms can be *technology-based* or *institution-based* (Hanger-Kopp et al. 2022). The former include, for example, supply-side economies of scale, that is to say, high initial investment that pays off by decreasing unit prices if production is increased or continued over a long time; demand-side economies of scale, such as the levee effect where assets accumulate in the protected area just behind a newly constructed levee; or learning and complementarity effects; in other words, the use of an established product tends to be upheld the more familiar one is with the product and the more embedded the product is with other established technologies. Institutional self-reinforcing mechanisms include political authority, where actor coalitions with significant influence and resources maintain a path that corresponds with their interests. Institutional density refers to paths that are deeply embedded in existing institutional structures and are thus tedious to disentangle and change. Collective goods require coordination and communication between multiple actors and may thus lock in paths as the rules and interests for managing collective goods are hard to change. While several self-reinforcing mechanisms are distinguished in the literature (for a comprehensive list, see Hanger-Kopp et al. 2022), in practice, these distinctions may be difficult to maintain. In FRM, specific self-reinforcing mechanisms appear as learning effects and interconnected management that privileges certain kinds of expertise (Parsons et al. 2019); and as sunk costs of flood defences, high transition costs because of narrow technical expertise, strongly formalised institutions and organisations and the perceived responsibility for FRM at the state level (Wiering et al. 2017). Adaptive expectations, that is, basing future decisions on the experience of past events assuming that risks will remain as they are, manifest in difficulties to design levees and reservoirs not just to the level of previous but to unprecedented flood events (Kreibich et al. 2022).

How to overcome path dependency is not explicitly addressed in the early path dependency literature, as path dependency is by definition the absence of change. *Niche experiments* may provide an entry point for changing persistent paths (Schot and Geels 2008; Smith and Raven 2012). These innovations typically focus on a solution towards a local problem without transferring to other communities (Seyfang and Smith 2007). Niche experiments can provoke radical changes within their current system; in practice, however, they may just achieve slow adjustments (Schot and Geels 2008; Smith and Raven 2012; Geels et al. 2016). Examples of niche experiments in FRM are multifunctional technical-mitigation measures, property-level flood risk adaptation measures, bottom-up citizen initiatives or nature-based solutions (Thaler et al. 2019; Seebauer et al. 2019; Raška et al. 2022; Schröter et al. 2022). To encourage niche experiments, the literature distinguishes between different factors that enable or delay them (Thaler et al. 2019) such as policy entrepreneurs as initiators and promoters of experiments, the use of policy windows after a disaster event or current institutional settings that encourage the development and deployment of experiments. Modes of governance highly influence these developments (Green 2017; Hartmann and Driessen 2017).

As these studies above illustrate, the majority of existing FRM case studies have a narrow perspective, feature only selected elements of path dependency and operate at the national level. In the present study, we comprehensively track all elements in the Ennstal and Aist case study regions.

## Method

### Austrian flood risk management system

The Austrian FRM is mainly defined within the Austrian Water Act (1959), Forest Act (1975) and Hydraulic Engineering Promotion Act (1986). As main actors on the national and regional level, the Water Authorities (BWW) are responsible for fluvial floods, whereas the Forest Engineering Service in Torrent and Avalanche Control (WLV) is responsible for mountain hazards, especially torrential floods. Both BWW and WLV are organised in regional administrative branches at the provincial and district level.

The Austrian FRM is organised within a federal state system (Thaler et al. 2016; Rauter et al. 2019). The federal system includes three political levels with different tasks and responsibilities: (1) the national level provides the general policy framework and a large part of the financial resources (up to 50% of the planning and implementation costs) for the realisation of structural and non-structural measures; (2) the provincial and district level is mainly responsible for the planning and implementation process of the measures and carries up to 30% of the costs; and (3) the local level, described in detail below. The sharing of tasks and responsibilities within the federal state system encourages a wide range of different regulations and strategies for how to deal with floods in the country.

Local authorities have a prominent position within the Austrian FRM system. Within them, the mayors of the respective municipalities have the main executive role. Local authorities are responsible for (1) designing local land-use plans with the requirement to avoid new buildings in high-risk areas (i.e. 30-year return period); (2) developing and deploying local flood emergency operations; (3) providing the financial resources for the maintenance of the implemented structural measures; (4) negotiating with private land owners to implement property-level measures or to mobilise their land for the realisation of structural measures; and (5) providing up to 20% of the costs of realising structural measures. The Austrian FRM system follows a bottom-up approach, which means that the local authorities call on the superordinate governance levels to initiate the planning and realisation process for FRM measures. These procedures only refer to the management of fluvial floods in creeks and rivers, however. Local authorities are solely responsible for managing surface runoff from pluvial heavy rain events, which are an increasing risk because of climate change and surface sealing.

### Case study description

We analyse two case studies: the Aist catchment in the province of Upper Austria and the Ennstal valley in the province of Styria. Both regions are located in rural areas characterised by dispersed settlements, a large number of agricultural businesses and commuting of most inhabitants to larger peri-urban or urban agglomerations within the larger region. Both regions are highly prone to various natural hazards, such as river and torrential flooding, and will be relatively more affected by climate change in the future than lowlands (Gobiet et al. 2014; Schneiderbauer et al. 2021). The largest flood events have occurred in the past 20 years. In the Aist catchment, the largest event occurred in August 2002, causing direct damage costing more than EUR 140 million (Habersack et al. 2012; Puchinger and Henle 2007). In addition, the region was affected by various surface runoff events in 2009, 2013 and 2017. The Ennstal region was affected by a series of torrential floods in 2010, 2012, 2013 and 2017. In particular, the 2017 event caused direct damages costing more than EUR 19.7 million in the municipality of Öblarn alone (Clar et al. 2021b). As a response to these events, both regions instigated various structural and non-structural measures.

### Data and analytical approach

We apply a mixed-method approach for triangulation and cross-checking from different perspectives, including document analysis, analysis of event and construction databases, and semi-structured interviews. The interviews complement the other analyses by eliciting background information and retrieving mental models of existing management paradigms. This approach allows comparison, control and confirmation of the collected data and the interpreted results, while avoiding narrow, oversimplifying explanations.

The document analysis includes reports on flood event documentation of FRM agencies and emergency services, policy and project documents, municipal newspapers and protocols, media reports and other written sources. Events mentioned in these sources were compiled, checked for consistency and plausibility and then compiled into a timeline from 1980 to 2020 depicting the sequence of flood events and changes in exposure, as well as FRM measures. We refer to structural measures as an umbrella term for technical-mitigation measures, for example, dams, dykes and technical flood storage. By contrast, non-structural measures comprise land-use planning, early warning, individual preparedness, emergency responses, training, insurance and similar.

We conducted 16 semi-structured interviews with mayors and representatives of public administration, including active as well as retired officials; interviewees recommended further relevant actors they knew in the region (snowballing method; full list of interviewees in the Appendix Table 1). In addition, a citizen initiative and a landholder company were interviewed to provide an external perspective. Interviewees were asked to validate and refine the timeline, map actor coalitions in local FRM and elaborate on why at specific points in time specific FRM options were chosen while others were postponed, discarded or neglected, and how the selected options set the stage for ensuing developments. Because interviewees spoke from their personal, possibly biased point of view, the responses of different interviewees addressing the same topic were contrasted and factual information given were verified in the document analysis. The interview guideline was designed to operationalise the constitutive elements of path dependency; principally whether sub-optimality can be observed, and which self-reinforcing mechanisms were (or still are) at play. Interviews took 1–2 h each and were conducted between December 2020 and May 2021, face-to-face, by phone or online, depending on current COVID-19 pandemic restrictions. Interview transcripts were subjected to qualitative content analysis, first conducting deductive coding along the defined path dependency elements, then extending the code system inductively to accommodate emergent aspects. In the results, we compare the perspectives of mayors versus the regional administration; here, regional administration comprises officials from the regional branches of the BWW and WLV authorities (see the “Austrian flood risk management system” section).

## Results

**Fig. 1:**
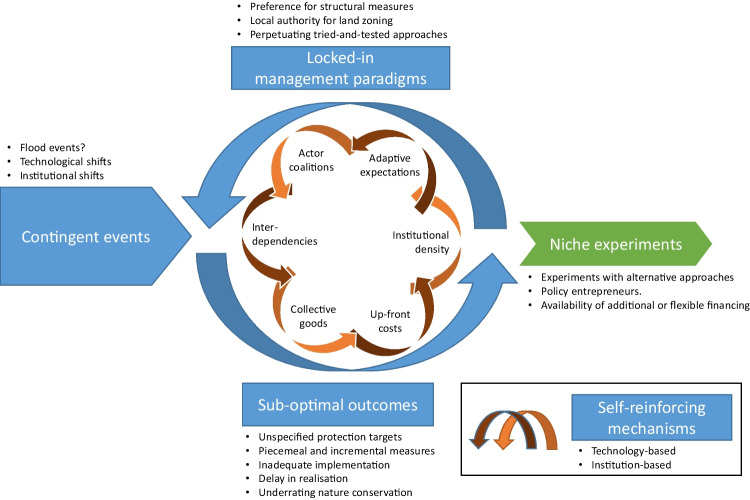
Characteristics and conditions of path dependency

Figure 1 illustrates the interplay of the constitutive elements of path dependency and assigns key aspects observed in the study regions to the respective elements. Locked-in management paradigms and sub-optimal outcomes promote each other in a path-dependent circle, advanced by self-reinforcing mechanisms. Contingent events instigate this circular process, whereas niche experiments may lead out of it.

### Locked-in management paradigms

#### Preference for structural measures

Starting with land drainage up to the 1970s and the straightening of river courses until the 2000s, the public administration in both study sites clearly preferred (and mostly still does) structural measures for reducing flood risks. Typical preferred measures are upstream technical flood storages in critical tributaries to buffer flood discharge, combined with linear flood dykes to channel runoff in peri-urban centres or at exposed premises and the dredging of riverbeds to maximise discharge cross-sections. When the administration plans several design variants of a given measure, the locations, but not the basic layout and construction, are varied. Structural measures are seen as a universal solution providing reliable and lasting protection from floods. The major dispute between residents and administration in the Aist region on the size and number of retention basins exemplifies how the administration’s management paradigm pushes for a narrow subset of measures. Citizens advocate a distributed network of many micro-scale retention measures (e.g. ponds, roadside ditches) to preserve the landscape and natural riverbanks. Mayors and regional administrations reject this approach, arguing that only a small number of large-scale basins is feasible because of fewer negotiations with property owners to obtain necessary land, better coverage of focal points in the catchment, lower total construction costs and easier maintenance of centralised structures. Because of continued citizen activism on the one hand and the administration’s insistence on large structures on the other hand, the Aist region remains to date in a deadlock situation where hardly any measures are built at all.

#### Local authority for land zoning

Zoning decisions taken decades ago still shape where FRM measures are necessary. Land zoning is conducted with the implicit intent of favouring short-term benefits in residential and commercial development while accepting long-term drawbacks in flood protection. Mayors maintain and defend their substantial leeway when deciding where to build in their municipality. Mayors who currently hold office typically cater to ad hoc demands of their constituents and the need to generate local tax revenues, downplaying the risks of building on the floodplain or designing FRM structures so as to reclaim areas for municipal development (i.e. levee effect). Mayors who are retired or have held their office for multiple terms look back critically on their own previous decisions, stating they were ignorant of and wishfully thinking about future risks in development zones. Representatives of superordinate governance levels call for more oversight by regional land-use planning; this critique, however, lacks political force to challenge the status of mayors as zoning authority.

#### Perpetuating tried-and-tested approaches

Implementing FRM measures follows a strict hierarchical and sequential process of homing in from a catchment-wide overview onto fine-grained local measures. This process complies with specified technical-hydrological criteria and is designed to narrow down the scope of options by stepwise discarding alternatives until the best variant remains. This ensures trustworthiness and accountability but is prone to reproducing established and undisputed strategies that have repeatedly passed this process.

### Sub-optimal outcomes

#### Unspecified protection targets

Providing a specific level of protection is a central aim of FRM; however, interviewees have vague notions of the protection they aim for. Regional administrations use the nationwide defined return period as default when designing structural measures, as the Hydraulic Engineering Promotion Act prescribes this return period for financing FRM measures from the budgets of national and regional authorities. This standardised return period is taken at face value by most interviewees. This default was not negotiated among local actors. In zones of increased vulnerability such as peri-urban centres, adding safety margins and freeboards up to doubles the protection level; this is, however, not made explicit in planning and financing documents. Regional administrations criticise mayors, sometimes pushed by their citizens, for opting for lower protection levels once they realise the sheer size and landscape impact of the measures designed to the standardised level. Mayors tend to take the last major flood event as reference for the desired protection level. Overall, mayors are prone to misperceiving return periods and flood zone demarcations. This causes many misunderstandings as regional administrators emphasise that flood return periods are not fixed numbers for the measure’s lifetime, but often fail to convey this caveat to mayors. Residents and mayors tend to ignore residual risk (i.e. the inverse of the protection target) and rather adhere to wishful thinking that the design default will suffice in future flood events. Stated protection targets do not extend beyond exposure to include other policy targets such as vulnerability or cost-efficiency.

#### Piecemeal and incremental measures

Mayors favour the implementation of small-scale structural measures. These quick-fix measures usually address the hot spots within the catchment like single buildings, bridges or drainage chokepoints. These measures are implemented ad hoc when opportunities or needs arise: as repair of damaged structures during flood recovery; as renovation of existing measures nearing the end of their lifetime; as stepwise upgrades if runoff and debris processes turn out differently than modelled; if adaption of other upstream or downstream measures requires subsequent refitting of existing measures. Usually, regional administrations provide operational support in planning and construction. The implementation of piecemeal and incremental measures demonstrates a process of maintenance and continuous improvement. This process becomes sub-optimal, however, if: measures are reactive, not proactive, or these measures create precedents or lock-in because they cannot be easily adapted or dismantled later, or stand-alone measures are not integrated into catchment-wide planning.

#### Inadequate implementation

Sub-optimal outcomes emerge when design schemes are watered down during construction or when idealised design assumptions neglect the conditions on the ground. It is mainly mayors who mention inadequate implementation since they have to cope when respective shortcomings become apparent during an emergency. Underdimensioning of measures occurs if land or funds can only be obtained for partial implementation, or if projections of settlement development or hydrological modelling used as design parameters are outdated by the time construction begins. Measures may be in need of repair earlier than expected because of inferior construction materials or erosion by the rough mountain climate. In some instances, built structures were designed for clear floodwaters and consequently underperform when the actual runoff carries hay bales, driftwood or debris, resulting in local blockages and overflow.

#### Delay in realisation

Many structural measures take years to decades from inception to completion. In the Ennstal region, the provincial court of audit finds that the realisation of structural measures takes 10–13 years on average. Multiple factors stall, protract or even derail the implementation process: fall-off in attention among mayors as other local concerns become more pressing; legal objections; revoking of finance commitments if the planning process spans several budget periods. Regional administrations highlight negotiations with private landowners as critical bottlenecks in project schedules. Often a flood event, such as the 2017 flood in Öblarn, can accelerate the implementation process and the availability of new financial resources. Thus, delay results in sub-optimality, as flood damages could have been avoided had measures been completed timely.

#### Underrating nature conservation

FRM decisions may be sub-optimal if they centre on the protection of humans and property and on economic interests but downplay nature conservation. Until the 2000s, land drainage and the straightening of river courses served to gain arable land and to construct hydropower plants. Public and private interests generally overruled nature conservation. This sub-optimal outcome has diminished in recent years, mainly due to the national transposition of the EU Water Framework Directive. Nature conservation representatives now have more standing in approval procedures, new structural measures must not worsen current ecological conditions and localised projects restore natural riverbanks and habitats for endangered species. The public administration still does not prioritise river ecology, for example in the management of alluvial sediments, however.

The interviewees do recognise that some past FRM decisions they were involved in turned out less optimal than they might have. They admit to ignorance, naivety or short-sightedness in themselves or their predecessors in office. They acknowledge technical limits to flood protection, in particular as climate change increases overall flood risk. Drawing on their experiences, they reflect on planning and technological options that were omitted or foregone but would have incurred more optimal outcomes had they been implemented, such as strict land zoning, upscaling successful small-scale experiments into the FRM mainstream or adopting a catchment-wide instead of a local scope in planning and decision-making.

### Contingent events

#### Flood events

Flood events do not qualify as contingent events in either the Ennstal or the Aist region, because they boost ready-made plans and prevalent risk reduction practices, but do not induce new strategies. Still, flood events function as the main impulse for overcoming standstill. Regarding technological impacts, flood events inform the performance assessment and possible upgrade needs of existing measures, direct attention to hot spots and allow re-calibrating of hydrological models with runoff observations. Regarding institutional impacts, flood events put FRM (back) on the political agenda, shape the protection targets, accelerate planning processes; facilitate access to (additional) national and regional funds, convince private landowners to provide land for measures or trigger inter-municipal collaborations. Flood events, in particular those with region-wide effects and millions of euro in damages, and those after quiet decades, structure all interviewees’ recollections and serve as markers in the collective flood narrative. Standing together during emergency and recovery builds the core of collective identity and social cohesion among local populaces. Flood events inspire awe and respect for the forces of nature; however, even the major flood events in the Ennstal region in 2002 and 2017 and the Aist region in 2002 and 2013 challenged but never overstretched FRM capabilities. Presumably, therefore, it will take even more severe disasters to initiate fundamental reorientation and an overcoming of the current path dependency.

#### Technological shifts: revised hazard maps, digital plan

Advances in hydrological modelling since the 2010s such as 2D modelling or topographical laser scans using aerial drones have produced revised hazard maps in both regions. These revised maps instigate a reassessment of land zoning and priority ranking of FRM measures. Revised hazard maps signal reorientation to a new path, as they prove the decisions taken on the previous path to be no longer viable. By the early 2000s, the Ennstal regional administration and its subcontracted civil engineers had shifted from hand-drawn plans duplicated by diazotype whiteprint to digital plans. The digital format increased accuracy of plot boundaries, freed up the administration workforce who had previously done the manual work of drawing and copying plans and facilitated data exchange between departments; however, the main benefit of digital plans lies in the easy planning and adaptation of multiple design variants. This enabled sincere citizen participation since objections or alternatives could now be easily integrated and compared. Previously, the regional administration was reluctant to accept any amendments as this meant elaborate manual re-drawing of plans.

#### Institutional shifts: EU directives, coordination projects

Regional administrations highlight how EU policy guides regional activities towards integrated water management. Soon after Austria’s accession to the EU in 1995, nature reserves on river plains were designated. The EU Water Framework Directive in 2000 and the Flood Directive in 2007 are major drivers for conservation of riverine life and revision of hazard maps. EU obligations lifted nature conservation from a side agenda to a cornerstone in approval procedures. The implementation of Areas of Potentially Significant Flood Risk (APSFRs), as prescribed in the Flood Directive, mandated regional administrations through the 2010s to compile hazard maps and thereby identify underprotected areas. EU funding played a central role during successive coordination efforts in the Ennstal region. Completed in 2008 after an extensive cross-departmental discussion process, the strategic Enns river guideline proposed recommendations for integrating management of river ecology, cultivated landscape, settlement development, tourism and flood protection. These recommendations formed the work plan of EU-co-funded LIFE (2005–2011) and LIFE + (2011–2015) projects that undertook extensive renaturation works including river widening and restoration of natural floodplains.

### Technology-based self-reinforcing mechanisms

#### Interdependencies/complementarity

means that the more technologies and their components depend on each other, the more embedded they become and the harder to change. Interdependency plays out along the same river or in the same catchment. To protect vulnerable regions downstream, structural measures are implemented in a staggered manner upstream. Measures are designed to complement each other, for instance, to catch floodwaters and alluvial debris as close as possible to their source, to collect runoff in channels with increasingly wide cross-sections, to provide sequential retention buffers or to divert river currents to wash up debris at selected locations where it can be removed easily. Management of upstream cropland, pastures and forests influences the effectiveness of downstream structural measures, as compacted soil retains less rainfall, or uprooted trees and washed-away hay cause blockages and additional damage downstream. This practice of daisy-chaining interlocking measures along the river course highlights the two-sided character of the interdependency mechanism: it helps to leverage synergies and to reach more optimal outcomes but necessitates adapting all elements if just one is changed.

#### High up-front costs

reinforce a path as economic reasoning suggests maintaining an expensive technology for as long as possible in order to recoup initial costs. Austrian FRM financing reinforces path dependency in both study regions in two ways: on the one hand, it delays the realisation of structural measures (see the “Sub-optimal outcomes” section) because small rural municipalities struggle to raise their legally required equity contribution to construction costs. On the other hand, as structural measures pass into municipal ownership upon completion, the municipalities have to carry all maintenance costs, for example for dredging alluvial sediments from retention basins or for servicing pumps and hatches. These maintenance costs heavily burden municipal finances and leave little budget available for alternative or innovative FRM activities.

#### Adaptive expectations

refer to sticking with a certain technology as long as it is deemed effective and sufficient. In the study regions, this mechanism appears in the ways that interviewees think that current protection conforms to future risk. The regional administration in the Ennstal region tends to discount major flood events as singular outliers, thereby justifying that current measures suffice and only need incremental adjustment. In addition, they argue that local projections of increased flood risk from climate change are too unreliable to be considered in measure design. Climate change is taken into account retrospectively because it gradually shifts the probability distribution of flood occurrence and thus recalibrates hydrological models and hazard maps, but not prospectively by assessing climate scenarios. By contrast, mayors in the Ennstal and the Aist region question earlier risk projections; experiencing repeated flood events makes them realise that previous expectations no longer hold. Regional administration and mayors agree that alluvial debris had previously not received the consideration it merits as a hazard process. Again, however, mayors but not the regional administration are concerned about heavy rainfall events as an upcoming hazard. This intersects with the self-reinforcing mechanism of institutional density (see the “Institution-based self-reinforcing mechanisms” section below), because the regional administration’s scope is formally restricted to flowing water bodies, whereas mayors have to cope with all remaining risks in their municipality.

Other technology-based mechanisms appear only sporadically. *Network and coordination effects* make it more attractive to use widespread mainstream technology; this mechanism appears when neighbouring municipalities or property owners share practical knowledge and access to flood risk zones via dirt roads. *Learning effects* describe how experience gained from using an existing technology makes it more attractive to use than switching to another; this mechanism appears when mayors transfer practical experiences from one small-scale piecemeal measure to the next, or regional administrations optimise construction processes.

### Institution-based self-reinforcing mechanisms

#### Collective goods or actions

rely on detailed rules stipulated by national and regional law and regulations. If these rules cannot manage conflicts, sub-optimal outcomes result. In our study regions, the collective goods mechanism manifests in the mobilisation of privately owned land and in upstream–downstream relations between the different local authorities. The building areas of almost all structural measures in the study regions require some privately owned land; the technical flood storages in the Aist region particularly rely on privately owned land. Thus, the property rights of the individual owners of this land (typically farmers and foresters) need to be balanced against the collective threat to downstream communities. Legally, the required plots could be obtained through an eminent domain procedure. However, to avoid political repercussions, mayors and regional administrations consider expropriation the last resort and instead strive for voluntary acceptance by purchasing the plots or otherwise compensating the owners. Extensive structural measures often require the joint agreement of several dozens of owners; single reluctant owners have substantial leverage and sometimes block the entire implementation process. Negotiating with owners often takes years and often reaches no consent. Consequently, availability of land is a recurrent theme in the interviews. Interviewees employ a range of strategies for mobilising land: preparing several design variants with different land requirements, customising measure design to the wishes of individual owners, exerting social pressure by disclosing opposed owners to the community or offering substitute plots instead of one-time payments. Some interviewees play the long game by patiently waiting until plots pass to heirs who are more open or by acquiring attractive plots in advance whenever an opportunity arises and retaining them as bargaining chips. In the Aist region, a citizen protest group convinced owners to hold back their plots, thereby effectively halting a building project.

To resolve collective conflicts in upstream–downstream relations, the mayors in the Aist region formed an inter-municipal water cooperative in 2007 to distribute costs and benefits fairly between upstream municipalities providing land and maintenance and downstream municipalities receiving protection. To date, however, this cooperative has implemented just a few measures. In the Ennstal region, problematic upstream–downstream relations appear in the clearing of creeks from washed-up debris. Mayors, as the representatives of downstream communities, are responsible for regularly inspecting creeks and, if necessary, prompt the respective owner to remove accumulated material. Mayors lack legal power to enforce compliance and eventually sanction owners, though; therefore, in most cases, debris remains in the riverbed until removed by volunteers such as the fire brigade or carried off by (hopefully small) floods.

#### Institutional density

describes the difficulty of changing a path once it is deeply embedded in institutional roles and procedures. The interviewees characterise FRM as a rigid process where mayors, various administrative departments and other stakeholders have legal standing only at specific pre-defined steps, for example at dedicated hearings or court proceedings. National funding conditions restrict the local room for manoeuvre. On the positive side, this strict process ensures certainty and equality before the law. On the negative side, the closed-off process makes it hard to adapt to new circumstances or to introduce innovative solutions. Measures are approved based on a cost–benefit analysis balancing the construction costs against the lives and assets protected. The cost–benefit analysis includes nature conservation, landscape and other non-monetised factors, but they play a more informative than pivotal role. Moreover, cost–benefit analyses may be stretched if there is strong political will to approve a specific project. In close-knit rural communities, the strict administrative process can be circumvented, as personal relationships transcend formal roles in the bureaucratic process or informal pre-checks align interests between mayors and various administrative departments, for instance, to tailor measures to available budget and land.

As a sub-optimal outcome of this self-reinforcing mechanism, high institutional density tempts some actors to pass on responsibility to others. This is most prevalent among mayors who voluntarily outsource local FRM decisions to the regional administration. Mayors justify their attitude by referring to the superior technical and legal knowledge of the administration’s expert officials; however, beyond this pretext, mayors mainly outsource to get rid of complex problems, to shift liability or to have a welcome excuse for unpopular decisions. The regional administration also occasionally passes the buck back to mayors (for achieving basic commitment among all local stakeholders) or to other administrative departments (particularly for jurisdictional reasons).

#### Political authority and actor coalitions

refer to (groups of) actors who use their power to uphold a path suitable to their interests. It intersects with the above mechanism: institutional density describes how the mere existence of a strict administrative process maintains a path, whereas political authority describes how those in charge use the administrative process to defend their position. Actor coalitions in FRM span a large network of administrative departments for water management, agriculture and forestry, transport infrastructure, nature conservation and others; politicians at all governance levels; civil engineering contractors; as well as large companies with premises in risk zones. When a new project comes up, the coalitions from previous projects are continued. All interviewees are well acquainted with the actor network and navigate its jurisdictions. The lower the governance level, the more the network interactions operate as long-standing personal relationships between individuals than as cooperation between institutions. Most actor coalitions follow the lines of administrative jurisdictions; if new coalitions emerge, these are formed at the local level. The Aist inter-municipal water cooperative, for example, developed guidelines for compensating land owners together with the chamber of agriculture.

The regional administration uses its central network position to defend its dominant role. The administration brings its legal, technical and bureaucratic edge to bear and aligns decisions with its interests through allocating or withholding funding, ranking on prioritisation lists or enforcing various guidelines. Concerned citizens are seen as challenging the administration’s authority. In some instances, the administration acts in a paternalistic manner, accommodating and analysing objections only to ultimately discard them. The administration mainly paints citizen initiatives as disrupting and derailing an otherwise well-established process. In turn, citizen activists form their own actor coalitions, reaching out to other concerned groups such as farmers and enlisting local politicians in their agenda. Thereby, the citizen initiative founded in 2011 in the Aist region still succeeds in delaying projects but cannot achieve the modification in measure design it wants. Interviewees from the regional administration claim that decision processes have become more transparent and inclusive since the 2000s. Today, all parties are regularly informed on the status and options in the implementation process. However, citizens and small landowners still suspect strategic withholding of information or backroom deals with civil engineering contractors and FRM funding agencies. By contrast, private actors with more power, such as industry or forest holdings, do not voice similar concerns and instead use their political influence and in-house legal and technical expertise to secure their seats in the actor coalition headed by the regional administration.

### Niche experiments

#### Experiments with alternative approaches

The current path is characterised by structural measures. Thus, niche activities experiment with a range of technological or management alternatives: small-scale built measures include obligating residents and companies to flood-proof their buildings and to install cisterns for pluvial retention on their properties; or adapting communal sewer piping. Nature-based solutions include adapting cropland and forest cultivation for better pluvial retention and less debris input, river widening and restoring side channels in selected locations, or fortifying riverbanks with natural materials such as boulders and tree trunks. Awareness-building activities include flood emergency training for residents. While some of these niche activities clearly go beyond Austrian state-of-practice, however, attempts at integrative management are still tentative. Only a few Aist municipalities prohibit settlement development in pluvial runoff zones; an initiative in 2007 for catchment-wide planning across the entire Aist region could not gain broad support. The Ennstal region started an integrated river management pilot in just 2021.

#### Policy entrepreneurs

Behind almost every niche development stands an influential and charismatic individual. They overcome institution-based self-reinforcing mechanisms through persistent lobbying at all levels and through recognising and taking advantage of loopholes in current funding and legislation. They excel in leveraging personal relationships to recruit a small circle of collaborators from diverse administrative and governmental positions. They have strong communication skills for mediating between opposing parties. Policy entrepreneurs are often mayors who already have a bridging function to other governance levels; some mayors can deploy additional political influence as members of the provincial or national parliament. In the Ennstal region, a (now retired) river supervisor implemented a range of river renaturation measures on his own authority because, until the 2010s, formal oversight was not yet strictly enforced. By contrast, in the Aist region, mayors and the regional administration describe the citizen initiative as an oppositional entrepreneur, hindering instead of enabling change.

#### Availability of additional or flexible financing

Policy entrepreneurs show substantial creativity in tapping alternative funding sources as national financing is tied to strict requirements. Alternative financing strategies include using recovery funds earmarked for flood repairs to rebuild stronger and better, diverting maintenance resources to incrementally upgrade measures, declaring labour and materials as in-kind equity slicing a structural measure into several small-scale measures to undercut funding ceilings, accessing regional development programmes such as Local Agenda 21, negotiating co-funding from road and railway providers with routes in the risk zone, selling excavated material to other construction works instead of paying for its disposal or, as already mentioned above, joining an inter-municipal water cooperative and accessing EU funding for LIFE projects.

## Discussion and conclusions

While previous case studies typically feature only selected elements of path dependency, the present paper comprehensively tracks all constitutive elements of path dependency for two Austrian case study regions. In doing so, we empirically apply the conceptual framework we recently suggested (Hanger-Kopp et al. 2022) and provide important lessons learned for mainstreaming the path dependency concept into the literature and practice of adaptation pathways.

### Operationalising the elements of path dependency

In the existing FRM literature, *lock-in* has predominantly been discussed in the context of grey infrastructure measures (e.g. Hübl and Kraus 2004; Wesselink 2016). Our analysis adds to the literature that describes lock-in beyond technological aspects, focusing on inertia in incumbent actor coalitions and individual mindsets (e.g. Tellman et al. 2018; Parsons et al. 2019). We find that the mental models of almost all interviewees reflect their enduring preconceptions of which options they generally consider effective and applicable in FRM. These preconceptions are in line with and sustain locked-in management paradigms, which reproduce the same FRM strategies and eventually culminate in potentially sub-optimal outcomes. These management paradigms maintain a state of general lock-in that endures as long as contingent events are absent.

Assessing whether outcomes are *sub-optimal* requires a counterfactual reference to what is deemed optimal, that is to say, in terms of a protection target or the tolerable level of residual risk (Hanger-Kopp et al. 2022). If decision-makers do not commit to clearly defined FRM targets, we cannot ascertain whether the sub-optimality criterion of path dependency applies. In our study regions, apart from a nationwide default protection target, interviewees do not agree on intended outcomes, which precludes any assessment of achieved outcomes and their (sub-)optimality. Still, we find that sub-optimality manifests in the ways that flood measures are implemented—too little, too late or with negative impacts on nature conservation.

*Contingent events* mark specific moments in time when FRM shifts from one path to another; however, Hanger-Kopp et al. (2022) emphasise the difficulties in extracting contingent events from historical changes in social, economic and political contexts without over-construing a particular moment or constellation as a turning point by neglecting the developments that lead up to it. Both rare flood events and technological or institutional changes could qualify as contingent events. Looking back in our study regions as far as the 1980s (which is as far as the availability of documents and the memories of interviewees allow), we cannot discern a contingent event or a narrow historical period when current FRM practices originated. Recurring floods did not fundamentally change the ways FRM is done but affected when and where measures were implemented; however, although they span longer periods, technological and institutional shifts in the past two decades have gradually reoriented FRM strategies.

Self-reinforcing mechanisms can explain why current paths are sustained. We find that *technology-based self-reinforcing mechanisms* are less prevalent in the study regions than institution-based self-reinforcing mechanisms, because there was little technological advancement or competition between products or designs during the observed timeframe. The construction basics of structural measures have hardly changed over recent decades. Even nature-based solutions, which entered FRM practice in the mid-2000s, draw on a limited portfolio of well-established techniques. As incumbent technologies were not threatened by upcoming niche innovations, there was comparatively less reason for deploying technology-based self-reinforcing mechanisms.

*Institution-based self-reinforcing mechanisms* are the prevailing reason why locked-in management paradigms persist and sub-optimal outcomes such as inadequate implementation or delay in realisation occur in the study regions. These mechanisms illustrate how those in charge defend their position, power and resources. Group thinking among a small circle of actors manifests in established actor coalitions combined with institutional density, leading to positive feedback and eventually to path dependency. We find instances, however, where these mechanisms are weaker, cut both ways or are navigated and circumvented. Institution-based self-reinforcing mechanisms tend to overlap and cannot easily be disentangled.

### Recommendations for overcoming path dependency

#### Encourage niche experiments

While established problem-solving strategies prevail in the study regions, we do observe some niche experiments undertaken by policy entrepreneurs that counteract self-reinforcing mechanisms and reorient selected FRM practices. These experiments do not qualify as contingent events, as they do not have an overarching impact on FRM strategies and their upscaling is prevented, for instance, by existing legal regulations. Nevertheless, niche experiments indicate potential turning points to leave dominant paths and should hence be encouraged by using any legal options at hand. Niche experiments cannot, however, substitute for more fundamental reforms, as they may easily reach their limits under intensifying climate change and current socio-economic developments.

#### Establish a stronger institutional link between FRM and climate change adaptation (CCA)

Many countries, including Austria, have a long and successful history of FRM to avoid, minimise and manage damage caused by floods. In parallel, CCA focuses on managing the risks resulting from climate change today and in the future; however, at all levels of Austrian governance, these two policy domains often operate in isolation (Schinko et al. 2016; Leitner et al. 2020). In our study regions, institution-based self-reinforcing mechanisms tend to keep these two policy domains apart. By contrast, international policy agendas such as the UNFCCC, the Sendai Framework or the UN Agenda 2030 ask for stronger cross-domain integration. To overcome these silos in risk governance, Leitner et al. (2020) propose to institutionalise a national climate risk council acting as an interface between FRM practice and political decision-making. Our findings support such a governance innovation as establishing a new institution could break up the existing group thinking among established actor coalitions.

#### Revise the national policy framework

Currently, default protection targets are defined at the national level, which pre-empts definition of anticipated optimal outcomes of FRM activities at the local level. Involving local communities could provide protection targets that are not only technologically and economically feasible but correspond better to local needs. National FRM policy prioritises technical-mitigation measures despite increasing knowledge that climate change exacerbates frequency, magnitude and community impacts of floods (Hanger-Kopp et al. 2022). The main reason for this prioritisation is that technical-mitigation measures are well known in the design, decision-making process, implementation and maintenance in comparison to other options, like nature-based solutions. However, this path dependency for technical measures will likely increase the risk of larger losses if FRM cannot recognise the increasing complexities within hydrological processes and risk reduction measures. Revised policy frameworks should also foster and facilitate inter-municipal water cooperatives and catchment-wide planning to overcome incumbent actor coalitions and institutional density. In the past, EU directives drove institutional shifts towards integrated water management. Thus, maintaining regulatory pressure from the EU policy landscape on the national policy framework could bring about further contingent events for switching to more optimal paths.

#### Improve professional training of administration officials

To realise the previous recommendations for changes in institutional and policy frameworks, substantial investments in human resources will be needed; moreover, improving the qualification of administration officials could accelerate the introduction of new knowledge into FRM practice. This challenge is even more problematic in many regions across the globe that are still limited due to scarcity of data or delay in adopting new technologies and models in FRM. Public administrations who lack the financial and human resources to detect and prevent path dependencies may be stuck with outdated information and techniques. Unless they are trained regularly, officials tend to reproduce the techniques learnt in their professional education decades ago, and new FRM knowledge only enters administrative practice when old employees retire and are replaced by new employees with current training. Additionally, using of new methods in FRM, such as adaptation pathways, storytelling or scenario-based forecast methods (Raymond et al. 2020), could constitute institutional and technological shifts, as in our case study, and ideally kick off an optimal path. International exchange and collaborations could help prevent path dependencies or even support leap-frogging.

Most elements of path dependency that we operationalised in this study appear similarly in both the Ennstal and Aist case studies, which speaks to the validity and generalisability of our results in the context of Austria, and presumably other western industrialised countries at risk of pluvial or fluvial flooding. Future research is needed to operationalise path dependency in CCA in a global South context. In less economically developed countries, elements of path dependency may impose constraints to adaptation that eventually lead to adaptation limits, that is, “the point at which an actor’s objectives (or system needs) cannot be secured from intolerable risks through adaptive actions” (Dow et al. 2013; IPCC 2019), much sooner than in the global North. It is thus crucial to understand the specific biophysical, economic, financial, human resource and governance and institutional constraints potentially leading to adaptation limits when operationalising elements of path dependency for the global South. Sub-optimality, for example, might shift from physical losses and damages to loss of human life and cultural heritage. In contrast to our case study, specific floods may become contingent events in countries with technologically and institutionally less developed recovery capacities. Institution-based self-reinforcing mechanisms can be expected to apply to all kinds of social organisation, even if they are less formal than in our case study. Our insights can be highly useful for developing more robust and realistic future adaptation pathways in other contexts by explicitly considering how the current situation has been predetermined by previous decisions.

## Data Availability

Data will be made available upon reasonable request.

## Appendix

**Table 1:** List of interviewees

No	Interviewee	Study region
1	Mayor of Municipality A	Ennstal
2	Mayor of Municipality B	Ennstal
3	Mayor of Municipality C	Ennstal
4	Water authority, provincial level	Ennstal
5	Water authority, district level	Ennstal
6	Water authority, district level, retired	Ennstal
7	Forest Engineering Service in Torrent and Avalanche Control, district level	Ennstal
8	Nature conservation authority, provincial level	Ennstal
9	Landholder company	Ennstal
10	Mayor of Municipality D	Aist
11	Mayor of Municipality E	Aist
12	Mayor of Municipality F	Aist
13	Water authority, district level	Aist
14	Forest Engineering Service in Torrent and Avalanche Control, provincial level	Aist
15	Inter-municipal water cooperative	Aist
16	Citizen initiative	Aist
